# Bioinspired Evolutionary Algorithm Based for Improving Network Coverage in Wireless Sensor Networks

**DOI:** 10.1155/2014/839486

**Published:** 2014-02-12

**Authors:** Mohammadjavad Abbasi, Muhammad Shafie Bin Abd Latiff, Hassan Chizari

**Affiliations:** Universiti Technologi Malaysia, Malaysia

## Abstract

Wireless sensor networks (WSNs) include sensor nodes in which each node is able to monitor the physical area and send collected information to the base station for further analysis. The important key of WSNs is detection and coverage of target area which is provided by random deployment. This paper reviews and addresses various area detection and coverage problems in sensor network. This paper organizes many scenarios for applying sensor node movement for improving network coverage based on bioinspired evolutionary algorithm and explains the concern and objective of controlling sensor node coverage. We discuss area coverage and target detection model by evolutionary algorithm.

## 1. Introduction

Wireless sensor network (WSN) has drawn a lot of attention in recent years. Developments of wireless sensor network enable them to operate with lower cost, lower power consumption, simpler computation, and better sensing of area when sensors move around. Furthermore, sensors also can sense the environment behind the movement, compute the data, and send the collected data to the sink node that can route the data to the other analyzing center through the internet [1].

Wireless sensor network has potential in many applications, such as healthcare, environment, industry, and environment monitoring surveillance in military, wildlife monitoring, and battle field. For instance, sensor network can be deployed in the environment for monitoring and controlling of plants and animal behavior [2] or in the ocean for controlling of temperature and seismic activities. However, in many places that are hostile, manual deployment is impossible and nodes have to be deployed randomly [3, 4].

The main problem in the wireless sensor network is deployment, coverage, and mobility strategy of sensor node; however, the coverage problem depends on a deployment sensor node in the wireless sensor network. There are some optimization methods which grow exponentially as the problem size increases. Therefore, an optimization technique that requires appropriate memory and computational process and yet produces great results is favorable, especially for implementation on sensor node. Bioinspired optimization techniques are computationally efficient alternatives to traditional analytical techniques.

Deployment of the sensor nodes can be placed randomly in a target area. When network size is large and sensor field is hostile, the only choice for deployment of nodes is to scatter with aircraft. However, when sensor nodes are scattered randomly, it is difficult to find best strategy for random deployment that could minimize the coverage hole and communication overhead. Minimizing of the coverage hole can improve the quality of service for sensor network [5, 6].

Recently, mobile sensor node has great impact on network coverage. They are equipped with vehicle and move around the area after random deployment to enhance network coverage. However, mobile sensor node is very expensive in comparison to the stationary node. It has maximum utility to increase the network coverage and lifetime and provide fault tolerance and quality service for network. The key objective for mobile node is to cover all area in the network and ensure each position has at least one sensor node for coverage. According to the monitoring area, three types of coverage have been identified: area coverage, target coverage, and barrier coverage. The mobile sensor node moves to exact location and connects to the other sensor node to form path coverage.

This paper presents how the mobility control can increase the coverage. In Section 2, we describe the model related to the sensing, coverage, and connectivity.  Section 3 describes the evolutionary algorithms for optimization coverage. In Section 4 the classification of mobility exploited coverage is described. In particular, a concept of the coverage holes is explained in detail.  Section 5 describes the dynamic optimization coverage using evolutionary algorithm with mobility to improve the network coverage.  Section 6 summarizes our contributions and research challenge in this open area.

## 2. Coverage Criteria

One of the fundamental issues in the wireless sensor network is achieving optimum coverage. The goal of optimum coverage in physical space is sensing area within sensing range covered at least with one sensor node. There are different criteria for the design coverage scheme based on different objective and application in WSN. This section is reviewing several models that have effect in WSN network.

### 2.1. Randomly Deployment Sensor Node

The main problem in the wireless sensor network is deployment of the sensor node; however, the coverage problem depends on a deployment sensor node in the wireless sensor network. Deployment of the sensor nodes can be placed randomly in a sensor field that is the only choice scattered with aircraft for deployment when network size is large and sensor field is hostile. However, randomly deployment where sensor nodes are scattered within the field (such as continues or grid) environment probability and probability from the aircraft exceptionality is needed [7].

### 2.2. Sensing Detection Model

There are two types of a sensor detection model: one is a unit-disk-based model and the other one is non-unit-disk-based model. Unit-disk-based model has fixed sensing range, which sensor node able to sense the environment inside the disk range. When useing sensor network as a unit graph, it is reasonable that the connectivity information of a graph contains sufficient information. Non-unit-disk based model has probabilistic sensing range, which sensing range is less than the distance.

### 2.3. Coverage Type

The key objective for mobile node is to maximize coverage in the network and ensure that area has at least one sensor node for coverage. According to the network to be covered, three types of coverage have been identified: area, target coverage, and barrier coverage. Area coverage addresses the problem of maximizing the detection rate in all spaces of sensing area with sensor node movement. Target coverage, on the other hand, moves a number of nodes to the specific point with the exact location for full coverage.

The main concern of barrier coverage is about finding the point in the path after deployment. Furthermore, mobile sensor node moves to exact location and connects to the other sensor node in the barrier coverage.

### 2.4. Fitness Function

Fitness function is a specific type of function that measures the optimality of a solution in evolutionary algorithm. Depending on the goals of the research, fitness function could be designed differently: single objective fitness function and multiobjective fitness function. In single objective fitness function, just one parameter for measuring the quality of the objective is used. With more than one objective, this entire independent objective should be combined, and this interaction is usually called hypostasis. All of above mentioned consist of objective about evaluating the solution in an evaluation algorithm.

### 2.5. Sensor Mobility

Sensor network used mobile node to enhance the coverage area. However, random deployment is not able to guarantee full coverage where a node is not in the exact position of sensing area. But, there are some mobility strategy to relocated sensor node in the exact position of sensing area after random deployment for improved network coverage. Mobility performance of the sensor has great effect on the sensor network to improve network QoS [8].

## 3. Evolutionary Algorithm

Evolutionary algorithms (EAs) are inspired from natural evaluation that helps to find optimum strategy for solving problem. The EAs continue a group of potential solutions to a problem. Hence, EAs use operator to create favorable potential solution. This operation is based on their optimal solution for problem. The EAs use this processes constantly to generate new population for optimal solution.

### 3.1. Particle Swarm Optimization

Particle swarm optimization (PSO) is relative of calculative models for outstanding by evolution. The purpose of PSO is responsibility to the best compound for problem under the presumption. PSO by Kennedy and Eberhart was first intended for simulating the social behavior. A difficulty optimized with the PSO is population of candidate solution. PSO model tagged particles and active these particles nearby in the search space similar to be common arithmetical formulae. Some fundamental concepts of particle swarm optimization that will be applied in bioinspired evaluation are described in the literature [9, 10]. The pursuits of the particle is aimed for establishing of best positions in the search space [11]. The particle swarm optimization for the *d*th dimension of position and velocity of *i*th particle is presented with following equation:

(1) V(t+1)=ω·v(t)+C1·(ld(t)−xd(t)) +C2·(gd(t−xd(t))),

(2) $$\begin{array}{c} xd\left(t+1\right)=xd\left(t\right)+vd\left(t+1\right), \end{array}$$

where *v*(*t*) is velocity for particle *i*, *xd*(*t*) is the distance to be moved by this particle from its current position, *xd*(*t*) is the current particle location, *ld*(*t*) is its best previous local location, and *gd*(*t*) is the best global position. *C*_1_ and *C*_2_ are positive constant parameters called acceleration coefficients. The inertia weigh, *ω*, is a user-specified parameter that controls, with *C*_1_ and *C*_2_, the impact of previous historical principles of particle velocities is based on its present one [12].

### 3.2. Genetic Algorithm

Genetic algorithm is a family of computational models inspired by evolution. These algorithms encode a potential solution to a specific problem on a simple chromosome-like data structure and apply recombination operators to these structures so as to preserve critical information. Some general steps are required to solve a problem with GA. Amongst them, there is a main component that is problem dependent, and it is chromosome design. Chromosome is a set of string, which consists of all the genes, and indicates a solution to the problem. Each string is sometimes referred to as a genotype or alternatively a chromosome [13]. Although at first chromosomes are generated randomly and they could not be the good answers, during each generation the overall fitness of them would be increased [14]. In the literature [15–17] are some basic concepts of genetic algorithm application that will be applied in bioinspired computation.

## 4. Mobility Exploited Coverage Classification

Many researchers have been able to develop mobility schemes for improving network coverage with high QoS. Based on deployment objective, mobility can generally classified into three major categories: repair the coverage hole, optimizing coverage, and event based coverage [8].

### 4.1. Repairing Coverage Hole

Coverage hole (some spots are not covered) may be happening when some mobile node is not located in the exact position after deployment. The main objective of using sensor node is to repair the coverage hole in sensing area with redeployment of sensor network [5].

### 4.2. Optimizing Coverage

Optimizing coverage objective is leverage mobility to reduce the node overlap and maximize the network coverage. In a random deployment network, some node in the area has overlaps with the other sensor node. Hence, mobile sensor node can move around sensing area and adjust their position in order to optimize the network coverage [6].

### 4.3. Event Based Coverage

The goal of event base coverage is improving the target coverage by using mobile nodes. Event coverage has limited lifetime and does not need to be longer coverage [18].

## 5. Optimization Coverage

The difficulty in the wireless sensor network is coverage; however, the coverage problem turns on the coverage model in the wireless sensor network. Coverage model can be vouching for the quality of service of sensing area and allocated in the large diversity of the application. In this section, we introduce how to estimate the network coverage hole and optimize the coverage area. Set of sensor nodes deployed in sensing area and coverage-estimate problem is to determine if all point in target area have *k*-coverage, where each point is at least covered with one sensor node. Optimization coverage problem is mostly studied in coverage optimization problem, while it also emphasizes the network lifetime and balanced energy consumption for *k*-coverage of sensor network with minimum mobility of sensor node.

Authors in [2] proposed P-BEEG algorithm which prolonged survival time of the sensor network, but all cluster heads can communicate with a base station, and the nodes are stationary in P-BEEG approach. The deployment method of sensors and optimizing movement strategy are developed for MSN, which taken into account the important issues and the key parameters about affecting energy consumption of nodes and maximizing the network lifetime in mobile sensor networks.

The authors in [19] proposed Optimization Movement control (OMC), for controlling of movement strategy in mobile sensor network. In the first deployment, *S* mobile node is randomly deployed and *F* node will be deployed in the rectangle grid with communication radius *R*; the hop of communication between *F* and *S* is one. The proposed algorithm works based on the evolutionary algorithms (SPSO) for optimal coverage in the mobile sensor network. The aim of this algorithm is to find particle position which is based on evaluation fitness function. The evaluation fitness function used simulated annealing (SA) and PSO. Simulated annealing is the combinational optimization and can help PSO algorithm to get high rate convergence and success in search space (SPSO). The SPSO is used for movement strategy; the algorithm accepted the new criteria that it helps fitness function to become worse in a limited area instead of extra criteria to accept the new optimal solution. The new criteria Δ*f* calculated the new particle position between two fitness functions where Δ*f* < *ε*. In evolutionary algorithm, fitness function has great effect in mobile sensor network. Also the author makes some improvement in PSO algorithm where velocity has magnitude director, which velocity have *X*-velocity and *Y*-velocity based on the following equation:

(3) vx(t+1)=ω·v(t)+C1·(ld(t)−Xxd(t)) +C2·(gd(t)−Xxd(t)),

(4) $$\begin{array}{c} v_{x}\left(t+1\right)=X_{x}d\left(t\right)+V_{x}d\left(t+1\right), \end{array}$$

(5) Vy(t+1)=ω·v(t)+C1·(ld(t)−Xyd(t)) +C2·(gd(t)−Xyd(t)),

(6) $$\begin{array}{c} V_{y}d\left(t+1\right)=X_{x}d\left(t\right)+V_{x}d\left(t+1\right). \end{array}$$

When nodes are deploying in monitoring area, the value calculated by (4) and (6) is not able to map to the corresponding sensor node. For corresponding sensor node, they use fitness function. The fitness function requires the following issue: first, consider additional energy; second, calculate the neighbor energy and then consider the surplus and the consumption of energy. As illustrated on the three considerations, the author used new fitness function to make reasonable model as follows:

(7) f(x)=a1Ec+a2eaveEmax⁡−Ec+a31n−1 ×∑i=1  i≠jnEc(i)∗1(2Ri)2+1,

where *a*_1_ + *a*_2_ + *a*_3_ = 1, *a*_1_, *a*_2_ and *a*_3_ are impact factor of node and their neighbor *E*_*c*_ is energy for current node and *E*_*c*(*i*)_ is equivalent energy for neighbor node. According to SPSO algorithm for movement control, at first, they initiate the stage to collect the statistic of *F* node position and then, based on fitness function, to calculate the speed and change the position of *F* mobile sensor node. Therefore, SPSO and rectangle grid for mobile sensor node guaranteed fundamental network topology and can improve network coverage.

Deployment is one issue in wireless sensor (WSN) while network consists of stationary and mobile node allows sensor network to enhance coverage by self-organization technique. Author in [20] proposed the parallel particle swarm optimization (PPSO) to enhance the coverage for large area. The mobile node will use PPSO to relocate them to find optimal deployment in large area for various coverage optimizations. Actually, mobile node deployment based PPSO is appropriate for finding optimum solution in contentious area which some position needs cooperative and dynamically can change their position based on environment requirement. They assumed that all nodes know their position and use detection range *r*_*d*_, dependability *r*_*t*_, and communication range. When area is in sensing range of *n* sensor node at time *t*, the area detection dependability can be computed as

(8) $$\begin{array}{c} R\left(t\right)=1-\overset{n}{\underset{i=1}{\prod }}\left(1-r_{i}\left(t\right)\right), \end{array}$$

where *r*_*i*_(*t*) is the dependability of *i*th sensor nodes.

After sensor node deployment based PSO in position *X*_*i*_ = *x*_*i*1_, *x*_*i*2_,…, *x*_*i*3_, coordinates of all mobile sensor nodes and associative objective are presented by detection of environment. The velocity of particle adjusts the granularity for tradeoff between speed and precision should be randomly change and calculate the local best and global best fitness correlated with new granularity for utiliz validity based on PSO equation. However, each node has minimum ability for huge computation based PSO. Therefore, authors used PPSO for deployment optimization which divided all detecting environment in *n* group and each group includes some intelligence sensor node. In random deployment, the coverage hole of each part is not equal, then mobile node is divided into *n* parts as *s*_*i*_ = (*s*_*i*_/∑*s*)∗*N*, where *s*_*i*_ is coverage hole and *N* is the sum of mobile sensor nodes. The sensor nodes are considered about neighbor node during optimization, because mobile node is intelligent and performs optimization independently. So, if the distance is less than detection range, the mobile node should be redeployed in monitoring area. Furthermore, PPSO algorithm has great effect in deployment optimization which can improve network coverage and connectivity performance in wireless sensor network according to detection ranges and the position of node. However, computational time of particle swarm optimization (PSO) will increase exponentially as the search space increases.

The main issue of coverage and target detection in wireless sensor network are dynamic deployment in wireless sensor network. The main problem for coverage balancing between local and global best position is coefficient for current speed of particle in next steps. Authors in [21] proposed three dynamic PSO algorithms that decrease the computation cost in sensor coverage. The first approach is PSO-LA which is used Learning Automata to adjust the searching method to continue the current route for particle. The proposed algorithm suppose mobile node equipped with Learning Automata that has two exploits, which will flow the best and continue your way. Choosing the flow as the best action means the use of best experience and team experience which has great effect on next iteration current particle velocity and present particle velocity is unnoticed. In this case, they use PSO equation for velocity and position update for particle. On the other hand, continue-your-way action has great effect on global search in unknown search space. In this algorithm, the mobile sensor node with minimum repetition and relocation moves around. In the next algorithm, PSO-LA and Learning Automata are guides each particle to move around search space. Allocation of learning automates helps each particle to make decision for moving around without considering the other particle. Then, Learning Automata uses two actions based on PSO algorithm for best global position and moves in search space with current velocity in the right way. In this algorithm, each particle uses previous phase to determine its action with minimum energy consumption. For both previous algorithms, that authors have proposed based on PSO algorithm, cyclic and zigzag movement were the major problem in long distance movement. However, cyclic and zigzag movement are the major problem based on PSO-LA algorithm in long movement. They use high energy consumption and computation time. To solve this problem, PSO-LA algorithm with logical movement, same as PSO-LA algorithm just in last step, has not zigzag movement. Therefore, logical movement method has best local and global search with high convergence rate and increases the network coverage and lifetime with minimum number of movements. In PSO-LA approach, PSO and Learning Automata are hybridized where velocity of particles is corrected by using the existing knowledge and the feedback from the real implementation of the approach. To improve the performance of the PSO-LA, improved PSO-LA approach is proposed, movement strategy of a node without an impact from the movement of other mobile nodes and based on the result learned from its previous step movement. In the third one, Improved PSO-LA with logical movement, sensors virtually change new positions by computing their target areas with the same procedure of the improved PSO-LA, but the real strategy movement of the sensor nodes only happens at the final round after last destinations are defined.

The goal of existing algorithms is best deployment, where coverage guaranteed the quality of service of the WSN. The PSO is responsible for maximizing coverage under the presumption as set of role. An adjustment problem includes a fitness function delineating in problem. The approach in [22] proposed coverage optimization based PSO and Voronoi diagram. Voronoi diagram is very useful in sampling model for coverage hole. Furthermore, this model can calculate coverage hole based on particle encoded. Voronoi diagram can be used for WSN deployment for *N* sensor *s*_1_, *s*_2_,…, *s*_*N*_, and put sensor nodes act as the site. The measure of a coverage holes is requires set of point. These sets of points include the voronoi diagram and have distributed point on the boundary of a polygon. A particle is encoded a final explanation which represents of the best location of sensor nodes. The location of sensor *i* is made clear by two coordinators (*x*_*i*_, *y*_*i*_). Particle is consider of an *N* sensor nodes and can determine the sensor nodes best location. The fitness function objective is minimizing coverage holes. The voronoi diagram measures the coverage hole as set of interest points. The interest point is vertex of the voronoi polygon which determines as voronoi diagram and number of spot consistently in a boundary of voronoi polygons. These interest points can help sensors to meet each other in the region of interest by pulling force. The proposed algorithm estimated the coverage hole as follow. Firstly, compute the interest point and distance of each nearby sensor node. After the calculation of the distance if distance (*d*) is larger than the sensing range (*r*_*s*_), then its shows there is a coverage hole around the interest point. Thus, the optimum coverage is a total coverage hole in interest of the region. The complexity computation of this fitness objective depends on the number of sensor nodes and the size of the grid. PSO-Voronoi algorithm helps to optimize the coverage with sensible computational time. The complexity computation of this fitness objective depends on the number of sensor nodes and size of the grid. PSO-Voronoi algorithm improves network coverage but ignores the time complexity of defining Voronoi polygons.

Multiobjective Genetic Algorithm (GA) is used to examine the optimization of WSN layout which are considered two competing objectives. Two competing objectives are consist of overall sensor coverage and the lifetime of the network [23]. During the coverage operational time, sensors move to form a uniformly distribute based on the execution of the approach at a destination. However, the computation of this algorithm is not minimum. Authors in [24] applied particle swarm optimization (PSO) algorithm to increase the 1-coverage in mobile sensor networks and to minimize cost by finding the optimum positions for cluster head based on a well-known energy model.

The aforementioned algorithms mainly consider 1-coverage optimization, which each point in monitoring region is covered by at least 1 sensor node. However, 1-coverage optimization is not capable of providing a uniform sensor distribution over the monitoring region. Any point in monitoring regain can be covered by *k*-coverage (*k* > 1) which *k* can be set of sensor nodes and the area of the monitored region.  *k*-coverage can improve the network performance, which is propitious to the maximum possible utilization of the available nodes and balancing the node energy consumption.

Finding the best position for sensor node is a favorable use of availability of the sensor nodes, increasing network lifetime and stability of sensor energy. In [25] authors proposed optimization deployment for *k*-coverage with minimum mobility. Hence, the random deployment does not guarantee full coverage and there is some vacancy. In [25] first nodes are randomly deployed, then the sensor nodes analyze the coverage hole with the following: *k*-coverage, coverage hole, and coverage vacancy. *k*-coverage where any point of sensing area should at least coverage with *k* > 1 sensor node (*k* is the number of sensors). Coverage hole where the monitoring area *i*, not monitored by any sensor node. Coverage vacancy where the monitored area *i* is covered by *n*_*i*_ < *k* sensor. At the end, algorithms calculated the total uncovered area. In such proposed algorithm there are *n* = *λS* homogeneous sensor in sensing area and each sensor used-unit-disk based covered *πr* ^2^. Based on randomly distribution, vacancy density *λ* is $\sqrt{k}/r^{2}\sqrt{2\pi^{3}}$ and the number of vacancy in *M* sensing area used by *λs* as *S* → *∞* combine with *λπ* ^2^. The authors applied PSO algorithm for optimization deployment with minimum mobility. The PSO algorithm determined each particle in the *d*-dimensional space as *X*_*i*_ = (*x*_*i*1_, *x*_*i*2_, *x*_*i*3_,…, *x*_*id*_) where *i* represents a number of particles and *d* is the dimension and the previous best position of particle is *P*_*i*_ = (*p*_*i*1_, *p*_*i*2_, *p*_*i*3_,…, *p*_*id*_) and velocity among the search space is *V*_*i*_ = (*v*_*i*1_, *v*_*i*2_, *v*_*i*3_,…, *v*_*id*_) and best position of particles can be updates by (1) and (2). The proposed algorithm shows reduced distance movement significantly with the unlimited model and the PSO algorithm. Also it can help to get high convergence rate and increase scalability of sensor network.

The coverage and lifetime are two important issues in mobile sensor network that ensure high quality service for sensor network. Authors in [26] proposed PSO algorithm for improving the coverage with maximum movement of sensor node. Finding optimal position is executed by updating particle velocity and position based on (1) and (2). The proposed algorithm used PSO method to find optimal position according to penalty based on fitness objective. The penalty factor can be found with fuzzy penalty. The algorithm uses voronoi diagram to get best coverage and time efficiency. Voronoi diagram is specified beforehand and for each sites there will be a corresponding region consisting of all points closer to that site than to any other. They used fitness function to evaluate the solution to encode coverage problem in particle. The objective is to minimize the coverage hole in wireless sensor network. The coverage hole is calculated by using voronoi diagram, which the sensor act as sites, if all polygon vertexes are covered by sensor node, then the region of interest is fully covered. Otherwise, coverage hole exists in sensing area. Therefore the fitness function of coverage hole can be calculated based on (9):

(9)  minimize: ∑coverage_hole

(10)  subject  to  dmov≥Dmax⁡,

where coverage gap is the place not fully covered by sensor node around the target area and *d*_mov_ is the maximum distance moved by sensor and *D*_max⁡_ is maximum distance that a sensor is allowed to move. Hence (2) can be rewritten with penalty function as follows:

(11) minimize:∑coverage_hole  size+γP(dmov)subject  to  Dmov≥Dmax⁡,

where *γ* is the value action variable and *p*_(*d*_mov_)_ is penalty function and suitable *p*_(*d*_mov_)_ function as *n*:

(12) $$\begin{array}{c} p_{\left(d_{\text{mov}}\right)}=\max\;\left(0,\left(p_{\left(d_{\text{mov}}\right)}-D_{\max}\right)\right). \end{array}$$

*p*_(*d*_mov_)_ is equal to zero as long as the limitation is submitted, but when the constraint is disobeyed, *p*_(*d*_mov_)_ is equal to some positive value. The fuzzy system uses penalty parameter, *γ*, based on the *d*_mov_ value. The following equation is determined to return the value of *γ*:

(13) $$\begin{array}{c} \gamma =\exp^{a}, \end{array}$$

where

(14) $$\begin{array}{c} a=\{\begin{array}{cc} 0 & \text{if}\;d_{\text{mov}}<D_{\max},\\ \frac{d_{\text{mov}}-D_{\max}}{\Delta } & \text{if}\;{D_{\max}<d}_{\text{mov}}<D_{\max}+\Delta ,\\ 2\left(\frac{d_{\text{mov}}-\left(D_{\max}+\Delta \right)}{D_{\text{ROI}}-\left(D_{\max}+\Delta \right)}\right) & \text{if}\;{D_{\max}+\Delta <d}_{\text{mov}}<D_{\text{ROI}}. \end{array} \end{array}$$

In proposed algorithm, first, sensor deployed is randomly in two-dimensional area and all sensors have similar sensing range when sensor node is homogeneous. In every step, maximum distance movement by sensor passed to the fuzzy system to calculate the new value of penalty parameter *γ* and the value passed to the PSO for fitness objective. This condition continues when one stopping condition happens. The proposed WSNPSO_con_ algorithm as method based on PSO improves sensor network coverage and minimize energy consumption but the complexity of computational for voronoi polygon is huge.

The approach in [27] works based on particle swarm optimization (PSO) to solve the movement coverage problem. The main objective in movement strategy is to decrease the distance between the neighboring nodes, thus increasing coverage in the network. The proposed algorithm does not consider the stationary nodes which are not able to change their initial positions. However, to minimize energy consumption and to decrease cost, stationary nodes are widely used in real applications.

Wireless sensor node is randomly deployed in grid filed. For evaluation of sensor deployment, sensor field can be two-dimensional grid and use probabilistic detection model. Dynamic deployment provides coverage and target detection for wireless sensor network. The proposed dynamic deployment algorithm is “with virtual force directed coevolutionary particle swarm optimization” (VFCPSO). The proposed algorithm use coevolutionary algorithm for dynamic deployment, because the PSO algorithm uses particle in search space to find optimal position. However, it is difficult for PSO to find solution in large search space and it also has some disadvantage; when some particles are closer to the optimal positions the other particles move away from the best position. In VFCPSO, best deployment of global search is accomplished by the hybrid CPSO algorithm for improving network deployment. At first, initialize the swarm as *Q* in *n*-dimensional, *Q* · *x*_*k*_ is recent location of particle *k*, *Q* · *y*_*k*_ is optimal local position of particle *k*, and $Q\cdot \tilde{y}$ is global optimal position of particle. And then, calculate the coverage area based on*f*(*b*(*k*, *p*_*k*_ · *x*_*i*_)). After evaluating the effective coverage area, by using (15) calculate the attractive and repulsive virtual force between sensor nodes. Perform PSO update on *p*_*k*_ using (1) and (2) and compute the virtual force of *i*th particle in *j*th dimension with the following equation:

(15) $$\begin{array}{c} g_{ij}=\{\begin{array}{cc} \frac{F_{x}^{\left(i,j/2\right)}}{F_{xy}^{\left(i,j/2\right)}}*\mathrm{Max}\;\text{step}*e^{F_{xy}^{-(-1/(i,j/2))}}, & j=1,3,5,\ldots ,2n-1,\\ \frac{F_{xy}^{\left(i,j/2\right)}}{F_{xy}^{\left(i,j/2\right)}}*\mathrm{Max}\;\text{step}*e^{F_{xy}^{-(-1/(i,j/2))}}, & j=2,4,6,\ldots ,2n, \end{array} \end{array}$$

where the superscript of each factor is the index of sensor and index of particle which is virtual force using coordinate of virtual force. In proposed algorithm the potential processing capability of multiple nodes may contribute to best optimization performance. However, for WSNs, the energy efficiency should be taken into account in the deployment.

The approach in [28] proposed 2.5D and studied PSO based coverage optimization for WSNs on digital elevation models (DEMs). To compute network coverage on DEMs, a method of computing individual sensor node coverage is introduced. The authors also proposed an improved algorithm based on dissipative particle swarm optimization (DPSO). The basic steps of PSO based coverage optimization are as follows.

**Step 1**

Randomly initialize the speed and position of each particle. The range of speed is [−*m*/2, *m*/2]^*n*^ and the range of position is [0, *m*]^*n*^. Compute the fitness value of each particle using (1). Set the position of each particle as its best position *p*_*i*_ and set the position of the particle having the best fitness as the group best position *p*_*g*_.

**Step 2**

Update the speed and position of each particle using (1) and (2).

**Step 3**

Compute the fitness value of each particle.

**Step 4**

Compare the fitness value of each particle with the fitness value of its best position *p*_*i*_. Set the current position of the particle as *p*_*i*_ if it has better fitness.

**Step 5**

Compare the fitness value of each particle with the fitness value of the group best position *p*_*g*_. Set the current position of the particle as *p*_*g*_ if it has better fitness.

**Step 6**

Stop if the maximum number of generations *G*_max⁡_ is reached and the optimized deployment is represented by *p*_*g*_; otherwise return to Step 2 and continue.

If the sensing radius of each node is *r*, then computing the coverage of a node requires *O*(*r* ^2^) time and computing the coverage of *n* nodes requires *O*(*nr* ^2^) time. It requires *O*(*n*) time to update the speed and position of a particle using (1) and (2) and *O*(*n*) time to mutate its position. If there are *p* particles, then it takes *O*(*p* *nr* ^2^) time to update them and compute their fitness values in each generation of the algorithm. Therefore, the time complexity of the algorithm is *O*(*G*_max⁡_ *p* *nr* ^2^). Authors introduced better sensor coverage with significantly minimum computational effort. The method involves significant energy consumption in broadcasting initial and final positions. It also necessitates algorithms for collision avoidance and localization.

## 6. Concluding Remarks

Scale and density of deployment and constraints in battery, storage device, bandwidth, and computational resources pose serious challenges to the developers of WSNs. Issues of the node deployment, coverage, and mobility are often formulated as optimization problems. Most optimization techniques suffer from slow or weak convergence to the optimal solutions. This calls for high performance optimization methods that produce high quality solutions by using minimum resources. Bioinspired algorithm has been a popular method applied to solve optimization problems in WSNs due to its simplicity, best solution, fast convergence, and minimum computational complexity. However, nature of bioinspired algorithm can forbid its use for real-time applications which need high speed, especially if optimization require to be carried out mostly. Bioinspired algorithms require large sizes of storage device, which may limit their implementation for resource. Literature has numerous successful WSN applications that utilized advantages of bioinspired algorithms.

In this paper, we surveyed recent contributions to the problem of improving network coverage by evolutionary algorithm. Network coverage is an important performance metric for various applications in WSNs. The concept of mobility as it can be used for wireless sensor networks is improving network coverage. However, traditional approach is used stationary node for improve network coverage based on schedule for control of activity in a best way. Hence, by control of mobile node the network coverage can significantly improve for wide performance application.

This paper organizes many scenarios for applying sensor node movement for improving network coverage based on evolutionary algorithm and explains the concern and objective of controling sensor node coverage. However, there are many kinds of coverage control algorithms that have been proposed for different coverage based on different sensing models. These new sensing models depend on more than one sensor node and also this new model require to call new node mobility control. Also there is another feature for node mobility and this objective is not only to improve network coverage but also to increase network lifetime and enhance data details timeline and reliability at the same time. Some future research work may take into account how to minimize energy consumption for those coverage's with holes and how to control sensor node movement strategy to heal network coverage and improve network lifetime. Furthermore, other issues such as an energy consumption model about mobile nodes and their moving strategy need to be taken into account in developing movement strategies.
